# Cell Line-Dependent Variability of Coordinate Expression of p75NTR and CRABP1 and Modulation of Effects of Fenretinide on Neuroblastoma Cells

**DOI:** 10.1155/2016/7568287

**Published:** 2015-12-30

**Authors:** Yaoli Pu Yang, Simeng Wang, Xingguo Li, Nina F. Schor

**Affiliations:** Department of Pediatrics, University of Rochester School of Medicine and Dentistry, Rochester, NY 14642, USA

## Abstract

Neuroblastoma is a childhood neural crest tumor. Fenretinide, a retinoic acid analogue, induces accumulation of mitochondrial reactive oxygen species and consequent apoptosis in neuroblastoma cells. The p75 neurotrophin receptor (p75NTR) enhances the antineuroblastoma cell efficacy of fenretinide *in vitro*. We examined the role of the retinoid binding protein, CRABP1, in p75NTR-mediated potentiation of the efficacy of fenretinide. Knockdown and overexpression, respectively, of either p75NTR or CRABP1 were effected in neuroblastoma cell lines using standard techniques. Expression was determined by qRT-PCR and confirmed at the protein level by Western blot. Metabolic viability was determined by Alamar blue assay. While protein content of CRABP1 correlated roughly with that of p75NTR in the three neuroblastoid or epithelioid human neuroblastoma cell lines studied, manipulation of p75NTR expression resulted in cell line-dependent, variable change in CRABP1 expression. Furthermore, in some cell lines, induced expression of CRABP1 in the absence of p75NTR did not alter cell sensitivity to fenretinide treatment. The effects of manipulation of p75NTR expression on CRABP1 expression and the effects of CRABP1 expression on fenretinide efficacy are therefore neuroblastoma cell line-dependent. Potentiation of the antineuroblastoma cell effects of fenretinide by p75NTR is not mediated solely through CRABP1.

## 1. Introduction

The p75 neurotrophin receptor (p75NTR) has been implicated in both development and disease and, in its interactions with its cognate ligands and binding partners, regulates cell fate [1]. The signaling pathways transduced by p75NTR are complex and each can lead to several alternative downstream outcomes dependent on cell type and environment. The p75NTR is a transmembrane cell surface receptor, the intracellular domain (p75ICD) of which is cleaved off and functions as a transcription factor to modulate protein expression. Parkhurst et al. [2] have shown that p75ICD can translocate to the nucleus, associate with the cyclin E1 promoter, and increase mRNA levels of cyclin E1 in PC12 and HEK293 cells. We have previously demonstrated that p75NTR affects cellular response to oxidative stress [3] and upregulates the enzymes that mediate cholesterol biosynthesis [4]. More recently, it was demonstrated that knocking down p75NTR expression in neuroblastoma cell lines attenuates the cellular response to the chemotherapeutic drug, fenretinide, while p75NTR overexpression has the opposite effect [5]. However, the mechanisms that underlie the potentiation of the effects of fenretinide by p75NTR are incompletely understood.

Neuroblastoma is the most common extracranial solid tumor of childhood. It derives from the neural crest and commonly presents clinically in the adrenal gland or sympathetic chain. Fenretinide, a retinoic acid derivative, is under active clinical investigation for the treatment of neuroblastoma. Unlike all-trans retinoic acid (ATRA), which is used to induce cellular differentiation in the treatment of cancer, fenretinide is known to cause apoptosis through generation of mitochondrial reactive oxygen species thought to “leak” from Complex II [5, 6] and differs structurally from ATRA by only a hydroxyphenyl group. In a recent phase II trial of 65 patients, fenretinide did not meet criteria for clinical efficacy [7] due to low bioavailability of the drug. However, there are now ongoing phase I clinical trials for a new formulation [8] as well as other drug delivery systems developing in the pipeline [9].

Our previous studies [5, 10] examined the dependence of fenretinide efficacy on components of the p75NTR proapoptotic signaling pathways and demonstrated enhancement of these pathways and attenuation of antiapoptotic pathways in the presence of p75NTR expression. A microarray study has identified CRABP1 as one of the 52 cancer-related genes of which p75NTR alters the expression [11]. The presently described studies test the hypothesis that p75NTR-induced potentiation of the effects of fenretinide on neuroblastoma cells occurs through regulation by p75NTR of the expression of the cellular retinoic acid binding protein I (CRABP1). The studies of others suggest that CRABP1 enhances conversion of fenretinide to its more potent metabolite, 4-oxo-fenretinide [12]. If this is true in neuroblastoma cells, this enhanced metabolism of fenretinide to 4-oxo-fenretinide could enhance the antineuroblastoma efficacy of fenretinide. Although little is known about CRABP1, it is implicated in regulating retinoid metabolism, rendering retinoids unavailable to nuclear receptors [13], and increasing intracellular concentrations of their active metabolites [14].

## 2. Methods

### 2.1. Cell Lines and Reagents

IMR-32, SH-SY5Y, SH-EP1, and SK-N-AS human neuroblastoma cells were obtained from the American Type Culture Collection (Rockville, MD). SH-EP1 cells were transfected to effect overexpression (p75OE cells) or mock transfected (OE Ctrl) as we have previously described [5, 10, 15].

### 2.2. Knockdown of p75NTR

p75NTR was knocked down in p75OE cells with siRNA [5] using Lipofectamine 2000 according to the manufacturer's instructions (Life Technologies, Chicago, IL). In addition, stable knockdown of p75NTR in SH-EP1 cells in their native state was performed by lentiviral transduction as we have previously described [5] using shRNA. The sequences of shRNA against p75NTR are NC-1, GAGGATCGGAGGCTTGTCA; NC-2, GGACAGAGTCTGGGTGTATTTATTT.

### 2.3. Knockdown of CRABP1

Transient knockdown of CRABP1 was performed by nucleofection with a Nucleofector Kit V and the corresponding programs for the Nucleofector II machine (Amaxa, Gaithersburg, MD). The target sequence of siRNA against CRABP1 is CGGCATTTGCACGGTTTCGAA.

### 2.4. Overexpression of CRABP1

The human CRABP1 cDNA was cloned into the pGIPZ CMV-MCS-IRES-tdTom-F vector and the lentivirus was produced in 293TN cells by the calcium phosphate method. SK-N-AS or SH-SY5Y cells were transduced with lentiviral particles and fluorescent cells sorted by gating for RFP positivity.

### 2.5. Northern and Western Blotting

Northern and Western blots were performed as we have previously described [5, 10]. In each relevant figure, a representative blot is shown of at least three independent cell preparations and blots performed. Where indicated on the figures, the optical densities of Western blot bands were determined using Image J software (National Institutes of Health, Bethesda, MD).

### 2.6. Effects of Fenretinide on Cellular Metabolic Viability

Fenretinide was applied to cells in culture at varying concentrations as noted in the text and figure legends. The Alamar blue assay was performed as we have described [5, 10] to discern the time course of cellular metabolic viability for each concentration of fenretinide. All determinations were performed in triplicate and, in each case, a representative study of at least three performed is shown. Plots depict the mean and SEM within a representative study and between-condition comparisons were deemed statistically significant (Student's *t*-test) if at the *P* ≤ 0.05 level.

## 3. Results

### 3.1. Correlation of p75NTR and CRABP1 Levels in Neuroblastoma Cell Lines

**Figure 1:**
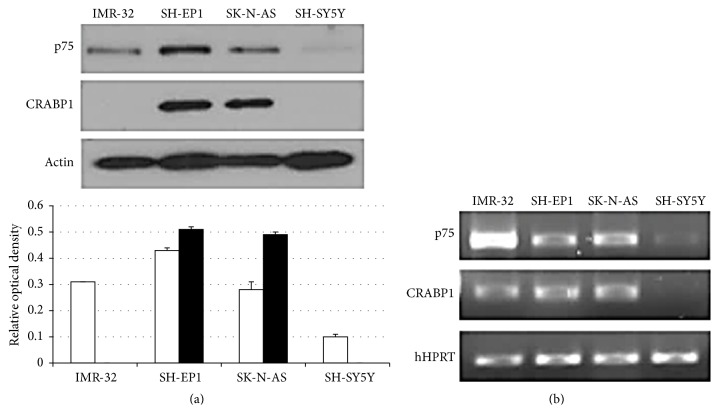
Western blot (a) and RT-PCR (b) for p75NTR, CRABP1, and loading marker protein and mRNA, respectively, performed on lysates of different neuroblastoma cell lines. Representative blots of 3 performed for each are shown, along with the mean and SEM relative to *β*-actin from three Western blots (a). hHPRT: human hypoxanthine-guanine phosphoribosyltransferase; open bars, p75NTR; solid bars, CRABP1.

If p75NTR expression affects fenretinide efficacy by inducing coordinate expression of CRABP1, then the two proteins should vary in concentration coordinately with one another. Formulation of this hypothesis followed our observation that p75NTR and CRABP1 protein levels vary coordinately with one another in neuroblastoid and epithelioid human neuroblastoma cell lines; the neuroblastoid line that has low p75NTR protein levels (SH-SY5Y) has undetectable levels of CRABP1 protein while epithelioid lines with higher p75NTR protein levels (SK-N-AS, SH-EP1) have higher levels of CRABP1 protein. IMR-32 is an “intermediate” neuroblastoma cell line with characteristics of neuroblastoma stem cells and mixed characteristics of neuroblastoid and epithelioid lines; IMR-32 cells have comparable levels of p75NTR protein to SK-N-AS cells. Although IMR-32 cells do not have detectable CRABP1 protein, they also express CRABP1 mRNA like SK-N-AS and SH-EP1 cells, suggesting CRABP1 mRNA expression appropriate to p75NTR expression with a block at the level of translation (Figure 1).

### 3.2. Regulation of CRABP1 Expression by p75NTR in SH-EP1 Cells

**Figure 2:**
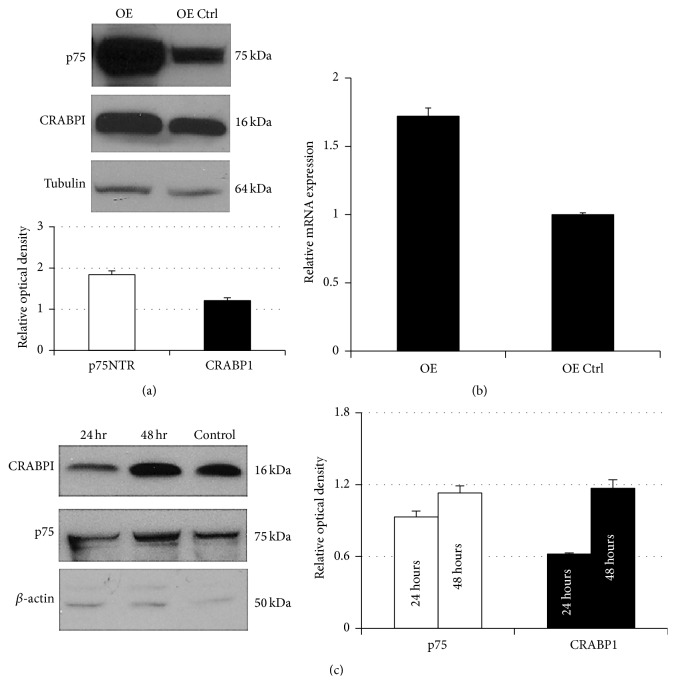
(a) Western blot for CRABP1 in SH-EP1 cells transfected with an expression construct for p75NTR (OE) or the analogous empty vector (OE Ctrl). Staining for *α*-tubulin serves as a loading control. The graph below the blot depicts mean optical density and SEM (*n* = 3) of each band relative to the optical density of the corresponding band for *α*-tubulin and normalized to OE Ctrl = 1.00. (b) qRT-PCR for CRABP1 performed on lysates from OE and OE Ctrl SH-EP1 cells (*n* = 3 independent samples each) demonstrates the overexpression of CRABP1 in OE cells relative to OE Ctrl cells (^*∗∗*^ *P* < 0.01, Student's *t*-test). (c) Transient knockdown of p75NTR with siRNA in SH-EP1 cells demonstrates coordinate regulation of p75NTR and CRABP1 expression (Western blot; *β*-actin serves as a loading control). The graph on the right of the blot depicts mean optical density and SEM (*n* = 3) of each band relative to the optical density of the corresponding band for *β*-actin and normalized to control = 1.00.

Our initial observation and cell signaling studies on p75NTR-induced potentiation of fenretinide efficacy were performed in SH-EP1 epithelioid human neuroblastoma cells [5, 10]. We therefore performed our studies of CRABP1 in this cell line. Commensurate with the coordinate variation of p75NTR and CRABP1 in native neuroblastoma cells, SH-EP1 cells induced to overexpress p75NTR (p75OE cells) had higher levels of CRABP1 protein than mock-transfected control cells (Figure 2(a)). This is supported by qRT-PCR data showing that the p75OE cells had higher levels of CRABP1 mRNA (Figure 2(b)).

Transient knockdown of p75NTR using siRNA resulted in transient knockdown of CRABP1 in the p75OE SH-EP1 cells, with levels returning to baseline upon recovery of p75NTR expression (Figure 2(c)).

### 3.3. Effects of Altered Expression of CRABP1 on Impairment of Cellular Metabolic Function by Fenretinide

**Figure 3:**
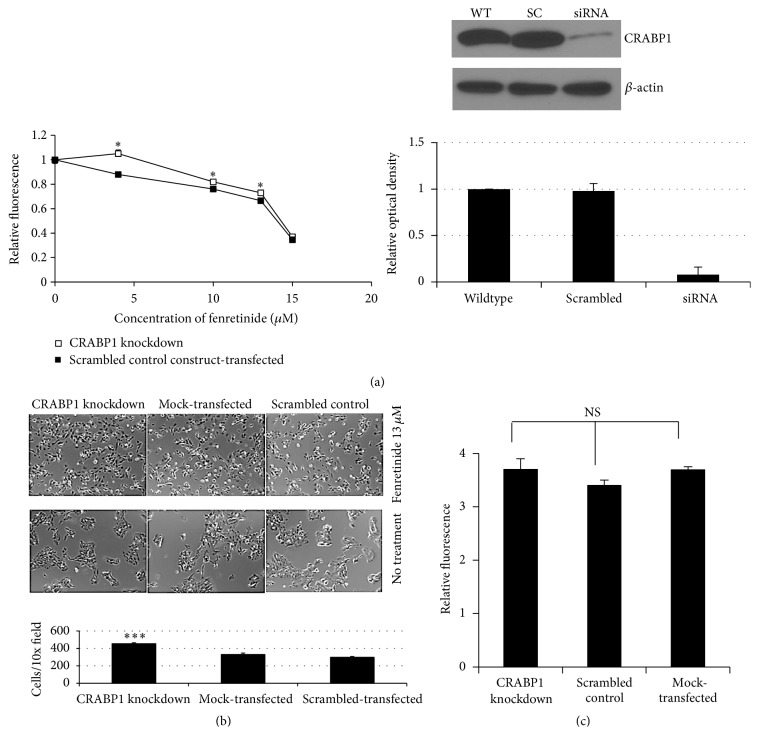
Metabolic viability and cell number of SH-EP1 cells transfected with CRABP1 siRNA or a scrambled control construct after treatment with fenretinide. (a) Alamar blue assay performed 60 h after treatment with fenretinide. Metabolic viability differs between CRABP1 knockdown (□) and scrambled control construct-transfected (■) cells (^*∗*^ *P* < 0.05) at 4, 10, and 13 *μ*M fenretinide. Note that Alamar blue assay does not account for cells already lost to apoptosis at the time of assay. A representative Western blot shows CRABP1 and *β*-actin proteins in Wildtype (WT), scrambled construct-treated (SC), and CRABP1 siRNA-treated (siRNA) cells. The graph below the blot depicts the mean optical density and SEM for 3 blots performed. (b) Representative photomicrographs (10x) of untreated and fenretinide-treated (13 *μ*M) CRABP1 knockdown, mock- (empty vector) transfected, and scrambled control construct-transfected SH-EP1 cells. As depicted in the graph, mock- and scrambled construct-treated cells showed greater cell loss (*P* < 0.001, Student's *t*-test; *n* = 3 determinations) than CRABP1 knockdown cells after fenretinide treatment. The three cell lines demonstrated equivalent cell culture growth and survival under control conditions. (c) Alamar blue metabolic viability assay demonstrates equivalent cell culture growth and redox reserve for the three cell lines under control conditions (NS: no statistically significant difference).

To assess whether manipulation of CRABP1 expression alone modulates fenretinide efficacy, CRABP1 was knocked down in native SH-EP1 cells using siRNA. Mock- and siRNA-transfected cells were then incubated for 60 h with 4, 10, 13, or 15 *μ*M fenretinide. The knockdown cells showed significantly greater metabolic viability after fenretinide treatment than mock-transfected controls (Figure 3(a)) and cell number (Figure 3(b)) in the absence of a difference in culture growth rate of untreated cells (Figure 3(c)).

### 3.4. Generalizability to Other Epithelioid Human Neuroblastoma Cell Lines of p75NTR-CRABP1 Coordinate Regulation as a Mechanism for p75NTR-Induced Potentiation of Fenretinide Efficacy

Demonstration of coordinate regulation of p75NTR and CRABP1, and CRABP1 knockdown-induced decrease in fenretinide efficacy in SH-EP1 cells caused us to examine whether these effects are also seen in the SK-N-AS epithelioid human neuroblastoma cell line. We previously demonstrated that SK-N-AS neuroblastoma cells exhibit the same p75NTR-induced enhancement of fenretinide efficacy as seen in SH-EP1 cells [5].

**Figure 4:**
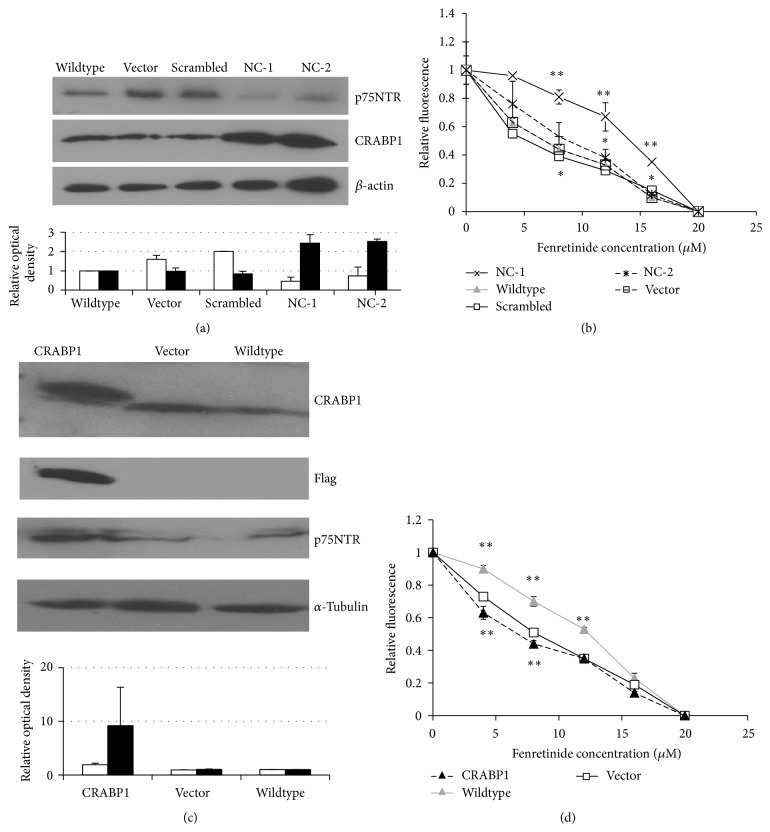
p75NTR, CRABP1, and response to fenretinide in SK-N-AS neuroblastoma cells. (a) Western blot of lysates from SK-N-AS cells in their native state (Wildtype), stably transfected with empty vector (Vector), a scrambled construct (Scr), or shRNA for p75NTR (clones NC-1 and NC-2). Knockdown of p75NTR is more efficient in NC-1 cells than in NC-2 cells. Blotting for *β*-actin serves as a loading control. The graph below the blot depicts the mean optical density and SEM for 3 blots performed. Open bars, p75NTR; solid bars, CRABP1 (b) Alamar blue assay of SK-N-AS cells treated as in (a) after treatment with fenretinide (*n* = 3 for each point; results for NC-1 (×, solid line; concentration required for growth inhibition by 50% [GI_50_] = 15) differ from those for Wildtype (gray triangle; GI_50_ = 7.5), Vector (□, solid line; GI_50_ = 5), and Scr (□, dashed line; GI_50_ = 6) with ^*∗∗*^ *P* < 0.01 and from those for NC-2 (×, dashed line; GI_50_ = 10) with ^*∗*^ *P* < 0.05; Student's *t*-test). Note that while NC-1 cells are more resistant to fenretinide than empty vector- and scrambled construct-transfected cells, NC-2 cells are not. Western blot (c) and Alamar blue assay (d) of SK-N-AS cells transfected with an expression construct for CRABP1 (CRABP1 (black triangle)) or an empty vector (Vector (□)) or examined in their native state (Wildtype (gray triangle)). Results for Wildtype differ from those for Vector (GI_50(Wildtype)_/GI_50(Vector)_ = 1.5; ^*∗∗*^ *P* < 0.01), indicating that transfection with an empty construct changes the fenretinide sensitivity of the cells; CRABP1 cells differ from Vector cells (GI_50(CRABP1)_/GI_50(Vector)_ = 0.8; ^*∗∗*^ *P* < 0.01) at 4 and 8 *μ*M fenretinide. The top band detected with anti-CRABP1 antibody in the CRABP1 lane is from Flag-CRABP1, the expression of which is induced. The graph below the blot depicts the mean optical density and SEM for 3 blots performed. Expression of p75NTR does not change significantly with induction of altered total expression of CRABP1 (Flag-CRABP1 + CRABP1). *α*-Tubulin is used as a loading control for Western blotting. Open bars, p75NTR; solid bars, CRABP1.

To our surprise, in contrast to the case for SH-EP1 cells, stable knockdown of p75NTR in native SK-N-AS cells resulted in upregulation of CRABP1 (Figure 4(a)). Furthermore, despite this upregulation of CRABP1, p75NTR knockdown resulted in resistance of SK-N-AS cells to fenretinide (Figure 4(b)), making it likely that the effects of p75NTR knockdown are not mediated through CRABP1 in SK-N-AS cells. In addition, induction of overexpression of CRABP1 without manipulation of or change in expression of p75NTR resulted in a less than 2-fold increase in fenretinide efficacy, statistically significant (*P* < 0.01) at 4 and 8 *μ*M, in SK-N-AS cells relative to empty vector-transfected cells (Figures 4(c) and 4(d)), suggesting that while CRABP1 is likely not the mediator of p75NTR-induced sensitivity to fenretinide, it may exert similar effects in SK-N-AS cells independent of p75NTR.

### 3.5. Effects of Manipulation of CRABP1 Expression on p75NTR Expression and Fenretinide Efficacy in Neuroblastoid Human Neuroblastoma Cell Lines

Although the p75NTR content of neuroblastoid neuroblastoma cells is generally lower than that of epithelioid neuroblastoma cells, p75NTR expression does enhance fenretinide efficacy in them, as well [5]. We therefore examined the effects of CRABP1 expression on p75NTR expression and fenretinide efficacy in neuroblastoid human neuroblastoma cells. SH-SY5Y cells do not express CRABP1 in their native state.

**Figure 5:**
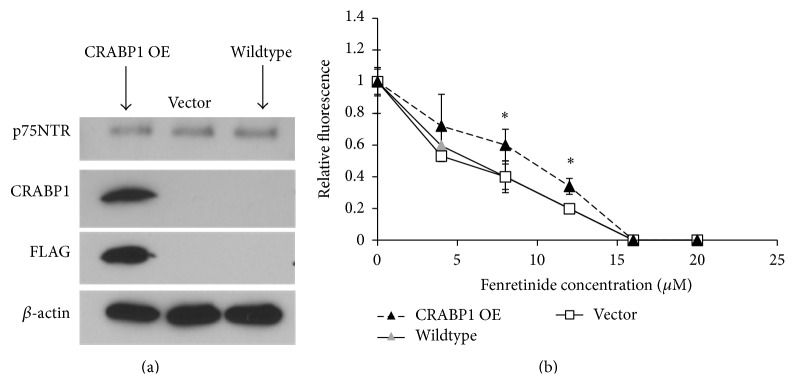
Western blot (a) and Alamar blue assay (b) of SH-SY5Y cells transfected with an expression construct for CRABP1 (CRABP1 OE (black triangle)) or an empty vector (Vector (□)) or examined in their native state (Wildtype (gray triangle)). Results for CRABP1 OE differ from those for Vector and Wildtype (GI_50(CRABP1)_/GI_50(Vector)_ = 2; GI_50(CRABP1)_/GI_50(Wildtype)_ = 2; ^*∗*^ *P* = 0.05) at 8 and 12 *μ*M fenretinide. *β*-Actin is used as a loading control for Western blotting.

Induction of expression of CRABP1 in SH-SY5Y cells did not alter expression of p75NTR (Figure 5). However, CRABP1-expressing SH-SY5Y cells are somewhat more resistant to the effects of fenretinide than empty vector-transfected or Wildtype cells. This is the opposite of the effect of CRABP1 overexpression in native p75NTR-expressing SK-N-AS cells.

## 4. Discussion

Fenretinide is a retinoic acid analogue originally developed in an attempt to improve the efficacy of retinoic acid as a differentiation-inducing agent for the treatment of retinoic acid receptor-positive cancers. While fenretinide was not, as it turns out, an inducer of differentiation or a particularly avid ligand for retinoic acid receptors, it did induce cancer cell apoptosis through induction of accumulation of reactive oxygen species in the mitochondria of these cells [6]. Phase II clinical studies of fenretinide that did not establish efficacy in the treatment of neuroblastoma [7], a common solid cancer of childhood that is frequently fatal within five years of diagnosis, have prompted efforts to enhance the bioavailability and efficacy of this drug* in vivo* [8, 9].

We have previously noted that expression by neuroblastoma cells of the p75NTR enhances the mitochondrial oxidative activity and cytocidal efficacy of fenretinide* in vitro* [5, 10]. Although the reactive oxygen species that accumulate in the mitochondria of cells treated with fenretinide appear to be generated at the level of Complex II [5, 6], p75NTR expression does not alter expression or activity of Complex II [16].

CRABP1 binds to retinoids and thereby sequesters them in the cytoplasm and prevents their shuttling to the nucleus. In so doing, it enhances the half-lives of retinoids in the cell. While fenretinide does not bind to CRABP1, its more active metabolite 4-oxo-fenretinide does [12]. We therefore hypothesized that induction of enhanced expression of p75NTR enhances the expression of CRABP1. From a therapeutic standpoint, we hoped that this enhancement of CRABP1 expression would increase the cytoplasmic concentration and mitochondrial redox effectiveness of 4-oxo-fenretinide after fenretinide administration.

Our results demonstrate neuroblastoma cell line-dependence of the effects of manipulation of p75NTR expression on CRABP1 expression and the effects of CRABP1 expression on fenretinide-induced cell death. CRABP1 protein concentration does covary with p75NTR protein concentration in native human neuroblastoma cell lines. Furthermore, our initial studies of SH-EP1 epithelioid neuroblastoma cells suggested that manipulating p75NTR expression leads to a coordinate change in CRABP1 expression, and manipulating CRABP1 expression alone (i.e., without manipulation of p75NTR expression) mimics the effects of p75NTR manipulation on fenretinide-induced cell death. This suggests that, at least in SH-EP1 cells, p75NTR could exert its effects on fenretinide efficacy through its regulation of the expression of CRABP1. However, our attempts to demonstrate these effects in another epithelioid line (SK-N-AS) and in a neuroblastoid line that was derived from the same parent line as SH-EP1 cells (SH-SY5Y) demonstrated, instead, the cell line-dependence of these phenomena.

The cell lines used were chosen for study because they collectively represent the gamut of histological and lineage subtypes of cells seen in neuroblastoma tumors. SH-SY5Y and SH-EP1 cells are both derived from the parent SK-N-SH cell line and represent, respectively, neuroblastoid and epithelioid sublines of that parent line. SK-N-AS is another epithelioid line unrelated to SH-EP1. Finally, IMR-32 is an intermediate line thought to represent neuroblastoma stem cells. While it is plausible that the redox transcriptome and consequent reaction to fenretinide of neuroblastoma cells would be dependent on their subtype, this appears not to be the case. Not only do SH-EP1 and SH-SY5Y cells behave differently from one another, SH-EP1 and SK-N-AS cells do as well, indicating that neither parental derivation nor lineage subtype predicts the redox transcriptome or the reaction to fenretinide of a neuroblastoma cell.

## 5. Conclusions

These studies underscore the complexity of neuroblastoma specifically and cancer in general as a therapeutic target. Even among cell lines of common lineage or tumor origin downstream signaling pathways and effects differ. In fact, there is ample evidence to suggest that, within a given patient's tumor at a particular moment in time, there are many genetically divergent subpopulations [17]. The fact that p75NTR and CRABP1 expression differentially affect one another and the impact of treatment with fenretinide in different neuroblastomas makes p75NTR or CRABP1, at best, complex biomarkers for likely responsiveness to that drug. Future studies must focus on downstream effectors in those neuroblastomas, like SH-EP1, in which coordinate regulation of these proteins leads to potentiation of the oxidative and cytocidal effects of fenretinide.
